# Where is the leak in the pipeline? Investigating gender differences in academic promotion at an academic medical centre

**DOI:** 10.1007/s40037-016-0263-7

**Published:** 2016-03-21

**Authors:** Jessica K. Paulus, Karen M. Switkowski, Geneve M. Allison, Molly Connors, Rachel J. Buchsbaum, Karen M. Freund, Deborah Blazey-Martin

**Affiliations:** Tufts Predictive Analytics and Comparative Effectiveness (PACE) Center, Tufts Medical Center, Boston, MA USA; Tufts Friedman School of Nutrition Science and Policy, Tufts University, Boston, MA USA; Division of Geographic Medicine and Infectious Diseases, Tufts Medical Center, Boston, MA USA; Colgate University, Hamilton, NY USA; Molecular Oncology Research Institute, Tufts Medical Center Boston, Boston, MA USA; Institute for Clinical Research and Health Policy Studies, Tufts Medical Center, Boston, MA USA; Division of Internal Medicine and Adult Primary Care, Tufts Medical Center, Boston, MA USA

**Keywords:** Faculty development, Academic promotion, Gender, Mentorship

## Abstract

**Background:**

Women are still under-represented in the senior ranks of academic medicine. As local surveys represent a critical initial step in addressing the challenges of gender disparities in academic promotion within institutions, we surveyed faculty at an academic medical centre to identify factors to improve the academic advancement of women.

**Methods:**

We conducted an electronic survey of all full-time faculty members in a Department of Medicine assessing academic rank and factors important in consideration for promotion.

**Results:**

106 faculty members (46 %) responded to the survey; 40 % of the respondents were women. There was a statistically significant gender gap in faculty rank (*p* = 0.002), with only 2 of 17 full professor positions occupied by women. Among faculty who had not yet requested promotion, women were more likely to report that they did not think an academic promotion would benefit them (69 vs. 32 % in men, *p* = 0.01), and to report a lack of encouragement for requesting promotion (50 vs. 29 %, *p* = 0.08).

**Conclusions:**

Targeting the perceived value of academic promotion among women faculty, increasing junior faculty mentorship and modifying annual review processes could address gender disparities in academic medicine ranks.

## Introduction

Gender inequalities in academic medicine faculty promotion have received significant attention over past decades [1]. With women comprising roughly half of medical school graduating classes, these disparities are now understood to reflect a ‘leaky pipeline,’ with women leaving academic medicine at higher rates than men [2]. Factors such as family responsibilities and engagement in activities not typically associated with academic productivity (i.e. teaching and patient care) [3–5] do not completely account for observed gender differences in academic rank [6, 7]. Moreover, reducing gender disparities in leadership has important implications for the success of both the individual and the institution [8], and the responsibility to fix the leaking pipeline must be shared by all, with consideration of how organizational structures and culture can be adapted to maximize gender equality [2]. Gender differences in how faculty interact with division leadership and mentors, and navigate the promotion process have been implicated in inequities in academic rank, [9, 10] and we hypothesized that they could represent barriers within our own institution that could be the target of policy changes.

To address the issue at one institution, we conducted a survey to identify modifiable factors that could be the focus of institutional interventions to reduce gender disparities in academic rank, and report on the steps taken by the institution in response.

## Methods

We conducted a cross-sectional survey of all current faculty members in the Department of Medicine at Tufts Medical Center in February 2012. Tufts Medical Center, the principal teaching hospital for the Tufts University School of Medicine, is a 415-bed academic medical centre located in Boston, Massachusetts. An electronic survey was developed by a subcommittee of the Women in Medicine and Science (WIMS) Committee at Tufts, which included a member of the Promotions Committee, and faculty at each academic rank. The survey design was informed by literature review and interviews with stakeholders in the promotions process (including the Department Chairman and Vice Chair of Clinical Affairs) to include possible barriers to promotion progress, and departmental processes that could be modified to improve the promotions process for all faculty. The survey was pilot-tested in a group of 10 faculty before distribution to the entire department. The survey included questions on demographic factors, current academic rank, length of time at rank, number of publications and whether faculty had formally requested consideration for promotion from their division chief. A series of questions assessed importance of factors in promotion consideration, on a 3-point Likert scale (‘somewhat’ or ‘very important’ versus ‘not important’). Additional closed-ended questions asked opinions regarding seven potential departmental processes that could enhance faculty promotion. Faculty received an email invitation to participate in the survey from the Chairman of the Department, and reminders one and two weeks after the original email invitation. Responses were compared between women and men using the Pearson Χ^2^ test and Wilcoxon rank-sum test. No personal identifiers were collected and analysis was conducted by an epidemiologist outside of the Department. This study was approved by the Institutional Review Board of Tufts Medical Center.

## Results

Of 232 faculty members in the Department of Medicine, 106 (46 %) responded to the survey; 44 % were women (Table 1). There was a statistically significant gender gap in rank, with women representing a majority of the faculty at the assistant professor rank (59 %, *n* = 29/49), but only 12 % (2 out of 17) of the faculty at full professor rank (*p* = 0.002).

**Table 1:** Characteristics of faculty survey respondents

Characteristic	Survey respondents (*n* = 106)^a^		All department faculty (*n* = 232)	
	Male	Female	Male	Female
*n* (%)	58 (60)	39 (40)	130 (56)	102 (44)
**Rank**				
Assistant professor, *n* (%)	20 (41)	29 (59)	50 (41)	71 (59)
Number of publications, median (range)	5 (0–67)	12 (0–49)		
Associate professor, *n* (%)	16 (67)	8 (33)	29 (65)	19 (35)
Number of publications, median (range)	23 (2–57)	23 (8–32)		
Professor, *n* (%)	15 (88)	2 (12)^b^	46 (97)	3 (6)
Number of publications, median (range)	155 (70–360)			
**Time related factors in years**, mean (SD)				
Age	51 (11)	45 (11)		
Time at Tufts Medical Center	16 (11)	10 (8)		
Time since completing degree	19 (11)	14 (11)		

^a^9 respondents did not provide gender information.^b^Not reported as only *n* = 2 female full professors responded to the survey.

Only 32 % (*n* = 13) of female faculty had requested consideration for promotion as compared with 49 % (*n* = 26) of males, though this difference was not statistically significant (*p* = 0.11). Adjustment for time since completing degree did not affect this finding (data not shown). Among survey participants who had not yet requested consideration for promotion, the majority (69 %) of women reported that they did not think an academic promotion would be of benefit to them, as compared with only 32 % of men (*p* = 0.01). More women than men felt that they had not met minimum time requirements for promotion (60 vs. 36 %, respectively, *p* = 0.08), and reported a lack of encouragement for requesting promotion (52 vs. 29 %, *p* = 0.08).

More than half of both men and women endorsed the following steps to improve the promotion process: requiring annual promotion reviews with each faculty member, including faculty promotion record as part of the division chiefs’ performance review, clarifying criteria for promotion, providing presentations on the promotion process (Table 2). With the exception of defining promotions criteria at hire, there were not significant gender differences in support for ideas for improving the promotions process.

**Table 2:** What could the department of medicine do to help facilitate academic promotion among faculty in general?

	Total *N* (%)	Males *N* (%)	Females *N* (%)	*P* value
Require division chiefs to hold individual annual meetings with each faculty member	71 (67)	37 (70)	33 (83)	0.16
Better define criteria for academic promotion at the time of hire	67 (63)	32 (60)	32 (80)	0.04
Consider faculty promotion record as part of that division chiefʼs annual review	61 (58)	32 (60)	27 (68)	0.48
Offer presentations on the process of academic promotion	56 (53)	32 (60)	23 (58)	0.78
Assign each faculty member a mentor at one academic rank higher	51 (48)	27 (51)	23 (58)	0.53
Provide protected time to work on the promotion application	45 (42)	21 (40)	21 (53)	0.22
Increase opportunities for participation in collaborative research groups	34 (32)	17 (32)	16 (40)	0.43

## Discussion

Similar to other institutions, we observed gender inequity across faculty ranks, despite the fact that men and women did not differ in terms of time at rank or publication productivity. Women disproportionately perceived that there was no personal benefit to promotion, and were less likely to report being encouraged to apply for promotion by department leadership. We also identified factors that men and women similarly felt would improve the promotions process, including standardizing relationships with mentors/chiefs, and presenting the promotion process more explicitly and accessibly.

While there is no ‘tenure clock’ at our institution, and promotion does not impact job security or salary for clinical faculty, women perceived the value of promotion differently than men. In fact*,* twice as many women as men in our study indicated that academic promotion held low value, similar to findings in another survey of medical school faculty [11]. While possible sources of gender differences in perceived value of promotion were not explored in our survey, they are likely the result of complex interactions between culture, academic environment, and psychological factors, such as the ‘confidence gap’ between men and women [12]. Roughly half as many women as men in our study indicated that they were encouraged to consider academic promotion, suggesting a potential need for improved mentorship and sponsorship. Strong mentorship and sponsorship are recognized as critical to academic career advancement, and are less available to women [9, 13, 14].

In collaboration with the Department of Medicine leadership, the Tufts WIMS Committee has promoted or implemented the priority interventions identified by survey respondents. As there were not dramatic gender differences in advocacy for possible interventions, the WIMS Committee prioritized the most popular responses overall. The WIMS Committee now holds approximately four events a year including hands-on workshops on the promotions process, as well as case discussions, lectures and informal social events designed to raise awareness about the career benefits of promotion, demystify the promotions process, and foster community and collaborations among faculty. WIMS subcommittee members (JKP, DBM) wrote a white paper summarizing survey results and recommendations for distribution to leadership stakeholders, and attended two division chief meetings to orally present these findings. The two main recommendations for department leadership were the development of institution-wide standardized mentorship protocols (with structured annual reviews which assess progress towards promotion and set goals for the coming year) and incorporating promotion rates as a metric of division chief mentorship performance. The WIMS Committee has also sponsored attendance at AAMC Women Faculty Professional Development Seminars for 5 Tufts women faculty. Though numbers are small, review of promotions data suggest improvement in gender gaps in rank: 9 women and 14 men were promoted in our department from 2007 to 2010 (4-year interval before WIMS committee launch), versus 18 women and 18 men from 2011 to 2014, the period in which the above interventions were launched.

Given the small numbers of faculty within strata defined by gender and rank, our study was limited by low statistical power to detect smaller differences between men and women. We attempted to limit the potential for selection bias by emphasizing respondent anonymity and privacy in communications soliciting survey participation. Overall, respondents were similar to the entire departmental faculty with respect to gender and rank, although we cannot exclude the possibility that respondents differed in other key ways, or assume that our results are generalizable to other academic medical centres.

Yet despite the relatively small sample at a single institution, our findings are similar to national data in detecting substantial gender gaps in academic rank, and possible drivers of this inequity related to mentorship support and the perceived value of promotion. Other institutions have also demonstrated that review of institution-specific data, followed by a targeted intervention, can increase gender equity [15]. Such local surveys represent a critical initial step in addressing the challenges of gender disparities in academic promotion.

## References

[1] Lautenberger D, Dandar V, Raezer C, et al. The state of women in academic medicine: the pipeline and pathways to leadership 2013–2014. Washington DC, 2014.

[2] Arnett DK (2015). Plugging the leaking pipeline: why men have a stake in the recruitment and retention of women in cardiovascular medicine and research. Circ Cardiovasc Qual Outcomes.

[3] Jolly S, Griffith KA, DeCastro R (2014). Gender differences in time spent on parenting and domestic responsibilities by high-achieving young physician-researchers. Ann Intern Med.

[4] Carr PL, Ash AS, Friedman RH (1998). Relation of family responsibilities and gender to the productivity and career satisfaction of medical faculty. Ann Intern Med.

[5] Kaplan SH, Sullivan LM, Dukes KA (1996). Sex differences in academic advancement. Results of a national study of pediatricians. N Engl J Med.

[6] Ash AS, Carr PL, Goldstein R (2004). Compensation and advancement of women in academic medicine: is there equity?. Ann Intern Med.

[7] Tesch BJ, Wood HM, Helwig AL (1995). Promotion of women physicians in academic medicine. Glass ceiling or sticky floor?. JAMA.

[8] Desvaux G, Devillard-Hoellinger S, Meaney MC (2008). A business case for women. McKinsey Q.

[9] Sambunjak D, Straus SE, Marusić A (2006). Mentoring in academic medicine: a systematic review. JAMA.

[10] Bickel J, Wara D, Atkinson BF (2002). Increasing women’s leadership in academic medicine: report of the AAMC Project Implementation Committee. Acad Med.

[11] Buckley LM, Sanders K, Shih M (2000). Obstacles to promotion? Values of women faculty about career success and recognition. Committee on the Status of Women and Minorities, Virginia Commonwealth University, Medical College of Virginia Campus. Acad Med.

[12] Kay K, Shipman C, The confidence gap. Atlantic. 2014.

[13] DeCastro R, Griffith KA, Ubel PA (2014). Mentoring and the career satisfaction of male and female academic medical faculty. Acad Med.

[14] Ibarra H, Carter NM, Silva C (2010). Why men still get more promotions than women. Harv Bus Rev.

[15] Fried LP, Francomano CA, MacDonald SM (1996). Career development for women in academic medicine: multiple interventions in a department of medicine. JAMA.

